# Salivary Xanthine Oxidase as a Potential Biomarker in Stroke Diagnostics

**DOI:** 10.3389/fimmu.2022.897413

**Published:** 2022-05-06

**Authors:** Mateusz Maciejczyk, Miłosz Nesterowicz, Anna Zalewska, Grzegorz Biedrzycki, Piotr Gerreth, Katarzyna Hojan, Karolina Gerreth

**Affiliations:** ^1^Department of Hygiene, Epidemiology and Ergonomics, Medical University of Bialystok, Bialystok, Poland; ^2^Students Scientific Club “Biochemistry of Civilization Diseases” at the Department of Hygiene, Epidemiology and Ergonomics, Medical University of Bialystok, Bialystok, Poland; ^3^Experimental Dentistry Laboratory, Medical University of Bialystok, Bialystok, Poland; ^4^Hospital Pharmacy, Provincial Hospital in Olsztyn, Olsztyn, Poland; ^5^Private Dental Practice, Poznan, Poland; ^6^Postgraduate Studies in Scientific Research Methodology, Poznan University of Medical Sciences, Poznan, Poland; ^7^Department of Occupational Therapy, Poznan University of Medical Sciences, Poznan, Poland; ^8^Department of Rehabilitation, Greater Poland Cancer Centre, Poznan, Poland; ^9^Department of Risk Group Dentistry, Chair of Pediatric Dentistry, Poznan University of Medical Sciences, Poznan, Poland

**Keywords:** stroke, saliva, xanthine oxidase, diagnostics, biomarkers

## Abstract

Stroke is one of the most common cerebrovascular diseases. Despite significant progress in understanding stroke pathogenesis, cases are still increasing. Thus, laboratory biomarkers of stroke are sought to allow rapid and non-invasive diagnostics. Ischemia-reperfusion injury is an inflammatory process with characteristic cellular changes leading to microvascular disruption. Several studies have shown that hyperactivation of xanthine oxidase (XO) is a major pathogenic factor contributing to brain dysfunction. Given the critical role of XO in stroke complications, this study aimed to evaluate the activity of the enzyme and its metabolic products in the saliva of stroke subjects. Thirty patients in the subacute phase of stroke were included in the study: 15 with hemorrhagic stroke and 15 with ischemic stroke. The control group consisted of 30 healthy subjects similar to the cerebral stroke patients regarding age, gender, and status of the periodontium, dentition, and oral hygiene. The number of individuals was determined *a priori* based on our previous experiment (power of the test = 0.8; α = 0.05). The study material was mixed non‐stimulated whole saliva (NWS) and stimulated saliva (SWS). We showed that activity, specific activity, and XO output were significantly higher in NWS of ischemic stroke patients than in hemorrhagic stroke and healthy controls. Hydrogen peroxide and uric acid levels were also considerably higher in NWS of ischemic stroke patients. Using receiver operating curve (ROC) analysis, we demonstrated that XO-specific activity in NWS distinguishes ischemic stroke from hemorrhagic stroke (AUC: 0.764) and controls (AUC: 0.973) with very high sensitivity and specificity. Saliva collection is stress-free, requires no specialized medical personnel, and allows continuous monitoring of the patient’s condition through non-invasive sampling multiple times per day. Salivary XO also differentiates with high accuracy (100%) and specificity (93.75%) between stroke patients with mild to moderate cognitive decline (AUC = 0.988). Thus, salivary XO assessment may be a potential screening tool for a comprehensive neuropsychological evaluation. To summarize, our study demonstrates the potential utility of salivary XO in the differential diagnosis of stroke.

## Introduction

Stroke is one of the most common cerebrovascular diseases. It is a sudden onset of focal or generalized brain dysfunction that lasts more than 24 hours and is caused by vascular disorders related to cerebral blood flow. Oxygen and glucose deprivation in the brain leads to decreased ATP synthesis and impaired synaptic conduction, causing neuronal malfunction and subsequent apoptosis or necrosis (1). The most common stroke causes include atherosclerotic lesions in extracranial and intracranial vessels, cardioembolism, carotid and vertebral artery dissections, hypercoagulable syndromes, and multiple systemic diseases. There are two types of strokes: ischemic (80%) and hemorrhagic (20%). Ischemic stroke is caused by closure/constriction of intracerebral vessels and hemodynamic abnormalities resulting in slowed cerebral flow, whereas hemorrhagic stroke occurs due to blood extravasation within the brain tissue (2). It is estimated that one person dies every 6 seconds due to stroke, which annually accounts for more than 5-6 million people worldwide. Thus, stroke is the third cause of death after heart disease and cancer (3). It is also the most common reason for permanent disability in people over 40 years old, with severe clinical, social, and economic consequences (4).

Many studies have shown that early revascularization treatment significantly improves patient prognosis (5–10). Although imaging studies such as CT, MRI, ultrasonography, arteriography, and echocardiography are the mainstay of the diagnosis, they may not be sufficient in some groups of stroke patients. Indeed, many other diseases have similar symptoms, making the differential diagnosis of stroke include brain tumors, migraine, epileptic seizure, hypoglycemia, hyperglycemia, hyponatremia, hypertensive encephalopathy, and hepatic encephalopathy (11–14). Not all patients are also treated at specialized stroke/neurology centers. Therefore, it is not surprising that rapid and non-invasive biomarkers of the disease are still being sought (15). Their source may be saliva containing numerous substances that pass from the brain into the blood (16–20). It is well known that the blood-brain barrier (BBB) is disrupted in stroke pathophysiology. The main factors damaging the BBB are mechanical failures or hypoxia damaging the cerebrovascular endothelium. These also include increased activity of matrix metalloproteinases (MMPs), enhanced secretion of cytokines, chemokines, and growth factors, and overproduction of reactive oxygen (ROS)/nitrogen (RNS) species by neuronal, glial, and immune cells. The damaged BBB becomes permeable to leukocytes inducing inflammatory processes and promoting the release of brain biomolecules into the blood (2, 21–29). Subsequently, circulating biomarkers can pass into saliva by passive, facilitated, or active diffusion, making saliva highly attractive in laboratory diagnostics. Saliva’s advantages are also evidenced by its easy availability, non-invasive and painless collection, and relatively high durability compared to blood and cerebrospinal fluid (16–18, 20, 30–35).

**Figure 1:**
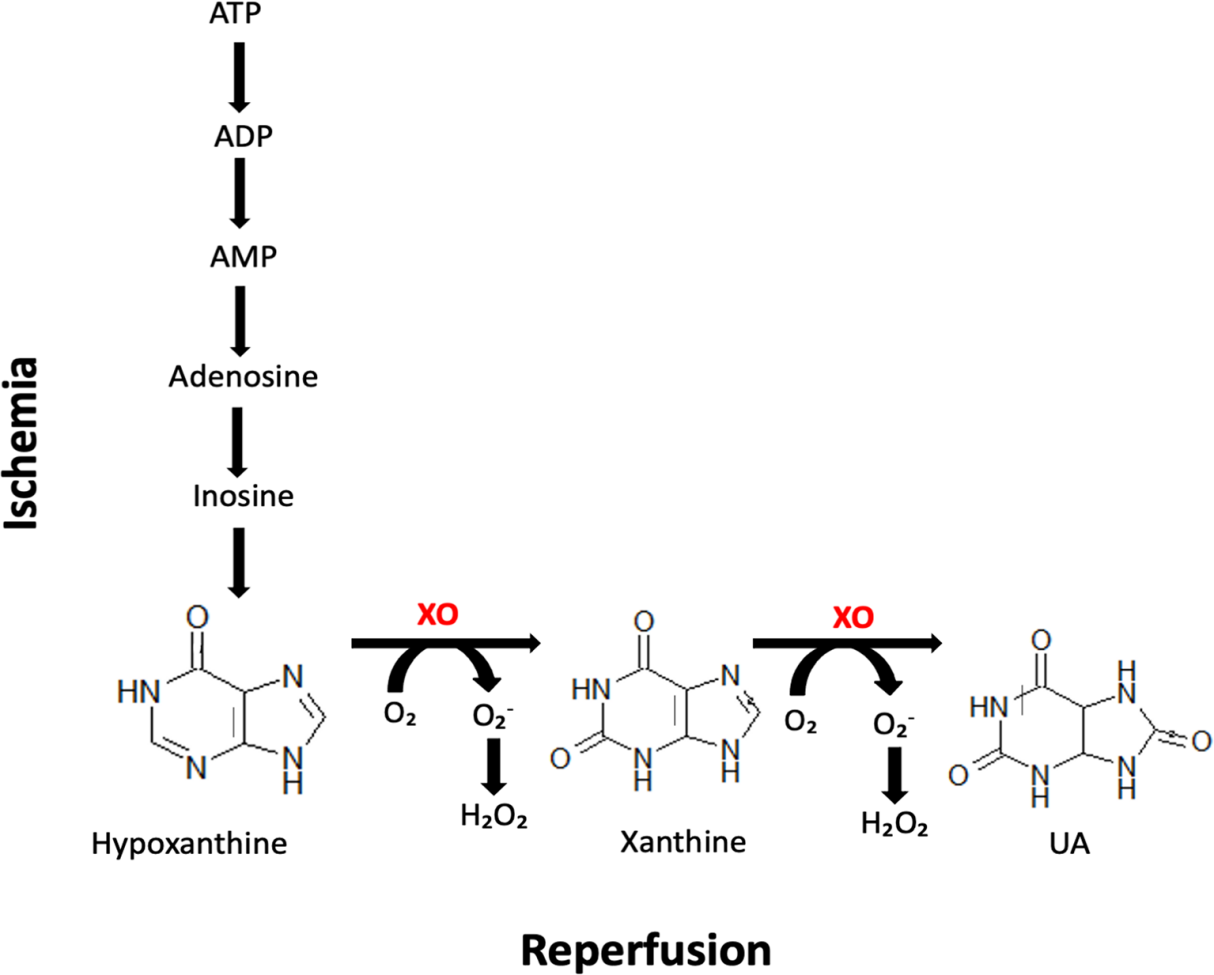
Biochemical reactions occurring in the stroke brain during ischemia and reperfusion. Xanthine oxidase (XO) catalyzes the conversion of hypoxanthine to xanthine and xanthine to uric acid (UA), generating high amounts of superoxide radicals and hydrogen peroxide (H_2_O_2_).

Recent studies suggest the potential use of saliva in ischemic stroke diagnosing or assessing the stress severity in stroke patients (36–38). However, there is a lack of non-invasive biomarkers to differentiate between different types of strokes. Given the critical role of xanthine oxidase (XO) in ischemic stroke pathomechanism (22, 24, 29, 39–41), we decided to compare the XO activity in saliva of patients with ischemic and hemorrhagic stroke. Ischemia-reperfusion injury is an inflammatory process with characteristic cellular changes leading to microvascular disruption. In ischemic stroke, ROS overproduction by XO hyperactivation is a major pathogenic factor contributing to brain dysfunction. XO catalyzes the conversion of hypoxanthine to xanthine and xanthine to uric acid (UA), generating high amounts of superoxide radicals, hydrogen peroxide (H_2_O_2_), and peroxynitrite (**Figure 1**). Higher formation of ROS/RNS in stroke brain results in neuronal oxidative stress responsible for altering the fluidity of biological membranes, modifying enzyme activity, uncoupling membrane transport, or deregulating membrane potential (24, 26, 29, 42–44). Although previous studies have not confirmed the clinical usefulness of serum/plasma XO in stroke diagnostics (45), evaluation of enzyme activity in saliva may bring new light to this issue. The results of many studies showed that saliva has the highest correlation with the brain, not the blood (46–48). When the BBB is breached, several glial, neuronal, and pericyte biomarkers appear in the blood, which then passes into saliva (22, 49–51). This is caused by the robust vascularization of the salivary glands allowing the efficient exchange of blood-derived components (52, 53). Since saliva can be collected non-invasively multiple times a day, it is a particularly attractive material for diagnosing cerebrovascular diseases (18, 32). Therefore, the present study aimed to compare XO activity and the enzyme products (e.g., hydrogen peroxide and UA) in non-stimulated and stimulated saliva of patients with hemorrhagic and ischemic stroke.

## Material and Methods

### Study Subjects

From June to September 2019, the research was carried out in the health center (Bonifraterskie Centrum Zdrowia) in Piaski–Marysin (Piaski, Poland). At this center, patients with numerous disorders, e.g., brain injury, spinal cord injury, vascular brain damage, polyneuropathy, myelopathy, sclerosis multiplex, surgically treated patients with a brain tumor, and those after cerebral stroke are hospitalized. Those individuals come from different provinces of the country.

The participation of each patient in the research was voluntary. According to its criteria, one experienced doctor, a specialist in neurorehabilitation, qualified all the subjects for the research.

Stroke patients in the subacute phase of the disease were included in the study group. All of the individuals were admitted to the neurorehabilitation unit immediately after the acute phase cessation and directly from the hospital. Each patient after the medical assessment was subjected to comprehensive individual and similar rehabilitation. It was established that during the time of the research, 385 patients were hospitalized in the neurorehabilitation ward due to different incidents, with 253 (65.71%) individuals who were stroke survivors. Most cerebral stroke individuals were able to cooperate, communicate, and understand instructions.

Data on the patients’ general health status and condition were obtained from their files and referred to gender, age, medications used, medical history, and time since diagnosis of cerebral stroke.

The functional status of the subjects was measured using the following scales:

1. Addenbrooke’s Cognitive Examination III (ACE III) for differentiation of patients with and without cognitive impairment (54).
2. The Barthel Index (BI) for measurement of individual’s everyday function, particularly performance in activities of daily living (ADL) (55).
3. The Berg Balance Scale (BBS) for determining a patient’s ability or inability to safely balance during a series of predetermined tasks (56).
4. The functional independence measure (FIM) explores the person’s social, psychological, and physical functioning (57).

Numerous patients (117 individuals - 30.39%) declined to participate in the study, including 34 (8.83%) subjects who did not come for saliva and examination sampling, even though they previously gave informed consent and were reminded from three to four times. Moreover, 48 patients (12.47%) were excluded from the analysis because they were uncooperative, i.e., they could not communicate and give conscious written informed consent for participation in the study. In addition, 3 (0.78%) patients were taken from the center to the other hospital because of deterioration of general health, 7 (1.82%) patients were not able for a sampling of the saliva because of general difficulties in understanding the procedure due to language and/or cognitive deficits, and 14 (3.64%) individuals abandoned the study after non‐stimulated saliva sampling due to psychological and/or physiological tiredness.

Therefore, ultimately, 30 (11.86% of cerebral stroke patients, i.e., 7.79% of all hospitalized individuals at the rehabilitation health center) completed the dental examination and saliva sampling and were considered in the analysis.

Most patients received the same meals at the center divided into a baseline diet for most individuals or a diet for diabetes mellitus patients. All the meals were prepared in the center and distributed to the patients every day at the same time.

### Study Criteria

The inclusion and exclusion criteria of cerebral stroke subjects to the study are presented in **Table 1**.

**Table 1:** Inclusion and exclusion criteria for the subjects in the study group.

Inclusion criteria	Exclusion criteria
confirmed cerebral hemorrhage or cerebral infarction based on magnetic resonance imaging (MRI) and computed tomography (CT)	unconfirmed cerebral infarction or cerebral hemorrhage based on magnetic resonance imaging (MRI) and computed tomography (CT)
good general condition	poor general condition
age of consent, i.e., over the age of 18 years	patients under the age of 18 years
no legal guardianship	legal guardianship
recovery from the acute phase of hemorrhagic or ischemic stroke in all brain areas	no recovery from the acute phase of hemorrhagic or ischemic stroke in all brain areas
the first admission to cure stroke unit was more than 5–6 (to 10) hours from the onset of the early neurological symptoms	the first admission to cure stroke unit was less than 5–6 hours from the onset of the early neurological symptoms and treated with thrombolysis
consciousness and giving of informed and written consent for a sampling of saliva and oral examination	unconsciousness and inability to give informed consent for saliva sampling and oral examination
adequate capacity to follow instructions, i.e., being able to answer questions during the study and understanding how to perform the procedures	inadequate capacity to follow instructions (insufficient cooperation due to cognitive and/or language deficits)
ability to gather a saliva sample	inability to gather a saliva sample
no stroke recurrence during the subacute phase	stroke recurrence during the subacute phase
ischemic stroke treated without thrombolysis or thrombectomy	ischemic stroke treated with thrombolysis or thrombectomy
patients without malnutrition (no weight loss over 10% during the previous three months or having body mass index higher than 18 kg/m^2^)	patients suffering from malnutrition (with weight loss over 10% during the previous three months or having body mass index lower than 18 kg/m^2^)
no heart failure (NYHA > II)	heart failure (NYHA < II)
no autoimmune disease (e.g., rheumatoid arthritis, systemic lupus erythematosus)	autoimmune disease (e.g., rheumatoid arthritis, systemic lupus erythematosus)
no psychiatric or cognitive disorders	psychiatric or cognitive disorders
no lung disease (chronic obstructive pulmonary disease) or cardiovascular disease (angina or uncontrolled hypertension)	lung disease (chronic obstructive pulmonary disease) or cardiovascular disease (angina or uncontrolled hypertension)
no XO inhibitors such as Allopurinol, Febuxostat, and Topiroxostat	XO inhibitors for the last three months
no vitamins and dietary supplements for the last three months	vitamins and dietary supplements for the last three months
non-smokers	smokers

### Control Group

The control group consisted of 30 generally healthy subjects similar to the cerebral stroke patients (study group) regarding age, gender, and status of the periodontium, dentition, and oral hygiene. This group included individuals reporting for dental examination to the Department of Restorative Dentistry of the Medical University of Bialystok (Bialystok, Poland) from March to September 2020. All individuals were provided with information concerning the research and gave their informed and written consent. Subjects used a regular and balanced diet (not restricted). They were also recommended to have routine physical activity.

### Sampling of Saliva

The study material was mixed non‐stimulated whole saliva (NWS) and stimulated saliva (SWS). The secretion of saliva was stimulated by utilizing 10 μL of 2% citric acid applied on the central part of the tongue every 30 seconds. Both types of saliva samples were gathered *via* spitting between 7:30 a.m. and 9:00 a.m. Before the dental examination, saliva sampling was performed in the health center in Piaski, from June to September 2019, i.e., during summertime, to keep similar weather conditions outside.

Before saliva gathering, patients were informed not to intake any solid and/or liquid food other than clean water at least two hours before saliva sampling. The individuals were also instructed not to have intensive physical activity for the preceding 12 hours and restrain to carry out any oral hygienic procedures (i.e., mouth rinsing, teeth brushing, gum chewing, etc.) (19, 58). All patients were in the subacute phase of stroke. Therefore, they had to take medicines regularly. However, the time from the last dose of any medication was minimally 2 hours before saliva sampling. In contrast, the subjects from the control group were not allowed to take any medication 8 hours before gathering saliva. Before sampling, the oral cavity was rinsed two times with distilled water at room temperature to avoid possible contamination from other sources. The saliva was collected in a separate, private room after a 5‐minute adaptation to the environment. The patients were situated in an adjustable chair that was individually adapted to the height of each individual. The subject’s head was slightly bent downwards and resting in a convenient position. Patients were asked to limit face and lips movements during the procedure (19, 58). The saliva samples were gathered into a sterile Falcon tube, and the amounts collected during the first minute were ejected. The NWS was accumulated for 10 minutes to avoid the individuals’ physiological and/or psychological tiredness, while SWS was gathered in the same manner for 5 minutes.

Afterward, the saliva volume was measured with a calibrated pipette (accuracy of 0.1 mL) (59). The salivary flow rate (SFR) of NWS and SWS was estimated by dividing their volume by the time necessary for the secretion, and finally, it was expressed in mL/min.

After sampling, the saliva was centrifuged (+4°C, 20 min, 3000 × g; MPW 351, MPW Med. Instruments, Warsaw, Poland), and butylated hydroxytoluene (BHT, Sigma‐Aldrich, Saint Louis, MO, USA) was added to the acquired supernatants, in the amount of 10 μL 0.5 M BHT in acetonitrile (ACN)/1 mL of saliva, to protect the samples from oxidation processes (60). Subsequently, saliva samples were situated in a container with dry ice, with a temperature of approximately –80°C, and stored for no more than three months for analysis.

### Examination of Oral Cavity

The oral examination was performed in a separate room, subsequently after sampling of saliva. The dentition was assessed in artificial light, using a plain mouth mirror and a dental probe, following the World Health Organization (WHO) criteria (61, 62). All accessible tooth surfaces were evaluated, and finally, they were scored as healthy, decayed (DT), extracted due to caries (MT), or filled because of caries (FT). The data collected were used in the calculation of the DMFT index (dental caries experience). It is the sum of DT, MT, and FT. The prevalence of dental caries was also determined. This index is calculated as a percentage of patients with DMFT > 0. Status of gingiva and oral hygiene was evaluated with the use of Gingival Index (GI) and Plaque Index (PlI), respectively, on the teeth, 16 (right permanent maxillary first molar), 12 (right permanent maxillary lateral incisor), 24 (left permanent maxillary first premolar), 36 (left permanent mandibular first molar), 32 (left permanent mandibular lateral incisor), and 44 (right permanent mandibular first premolar), using 4-degree scales (with scores from 0 to 3) (63). Before examining subjects’ oral cavity, calibration and training of two researchers who are dentists (P.G. and K.G.) was done by another experienced dental specialist (A.Z.). The intra‐examiner and inter‐examiner agreement for PlI and GI was assessed by another dental examination in ten subjects (κ >0.9). The dental evaluation was carried out subsequently after NWS and SWS whole saliva collection.

### Consent of the Bioethics Committee and Patients

All subjects, i.e., stroke patients (study group) and healthy individuals (control group), received written information on the study’s purpose, benefits, risks, and procedures. Full written and informed consent was obtained from all subjects following the Declaration of Helsinki for saliva sampling and dental examination.

The research was also approved by the Bioethics Committee of the Poznan University of Medical Sciences (resolutions 59/19 and 890/19).

### XO Assays

The activity of salivary XO (EC 1.17.3.2) was estimated using two biochemical methods. Firstly, the enzyme activity was measured colorimetrically based on the XO-catalyzed conversion of xanthine into UA. XO activity was calculated by the rise in absorbance at 293 nm. The activity of the enzyme was measured in pmol of UA/h/mL (64, 65). Secondly, XO activity was assessed fluorometrically using a commercial Amplex^®^ Red Xanthine/Xanthine Oxidase Assay Kit (Invitrogen, Waltham, MA, USA). In this method, XO catalyzes the oxidation of xanthine/hypoxanthine to superoxide radical and UA. Superoxide anion is then reduced to H_2_O_2_, which reacts 1:1 with Amplex Red reagent. The reaction occurs with horseradish peroxidase (HRP) and produces red-fluorescent compound resorufin. The absorbance of resorufin was measured at 540/590 nm wavelength. One unit of XO activity was assumed as 1 µmol of UA formed from hypoxanthine at 25°C.

The 96-well microplate reader BioTek Synergy H1 (Winooski, VT, USA) was used to measure the sample’s absorbance and fluorescence. All determinations were performed in triplicate samples. The results were presented as enzyme activity (μU/mL), specific activity (nU/mg protein), and salivary output (μU/min). Total protein content (TPC) was assayed colorimetrically using bicinchoninic acid (BCA) assay with bovine serum albumin (BSA) as a standard (Thermo Scientific PIERCE BCA Protein Assay Kit, Rockford, IL, USA).

### XO Products

To assess hydrogen peroxide (H_2_O_2_) concentration, Amplex^®^ Red Hydrogen Peroxide/Peroxidase Assay Kit (Invitrogen, Waltham, MA, USA) was used. The Amplex^®^ Red reagent reacts stoichiometrically with H_2_O_2_. The product of H_2_O_2_ oxidation is the fluorescent resorufin, which was measured at 540/590 nm.

UA concentration was estimated by the colorimetric method. Commercial QuantiChrom™ Uric Acid Assay Kit (BioAssay Systems, Hayward, CA, USA) was used. In this assay, iron reacts with UA and 2,4,6-tripyridyl-s-triazine generating a blue-colored complex. UA level is directly proportional to the intensiveness of the samples’ color estimated at 590 nm.

All determinations were performed in triplicate samples. The results were presented as nmol/L (H_2_O_2_) or μmol/L (UA).

### Statistical Analysis

Sample size calculation was assumed *a priori* based on our anterior clinical study. For this purpose, the online sample size calculator *ClinCalc* was utilized. The level of statistical significance was determined on 0.05, and the power of the study was 0.8. XO specific activity and UA level in NWS were used for analysis. Patients’ minimal number amounted to 12 (per one group).

In order to assess the inter-and intracorporeal agreement of dental examinators, the online *Cohen Kappa* calculator was used.

Statistical analysis was conducted using GraphPad Prism 8 for macOS (Graph- Pad Software, La Jolla, CA, USA). The examined variable distribution was assessed by the Kolmogorov–Smirnov test, while the homogeneity of variance was by Levene’s test. The student’s t-test was used to compare two groups, while analysis of variance (ANOVA) with Tukey’s *post hoc* test was performed to compare three groups. The defined statistical significance was p < 0.05. p-Values were computed with correction for multiple comparisons. The results were expressed as mean ± standard deviation (SD) and 95% confidence intervals (95% CI). Correlations between clinical data and salivary biomarkers were carried out with the Pearson correlation coefficient. Multivariate analysis of the simultaneous impacts of many independent variables on one quantitative dependent variable was made using linear regression. Stroke type, ACE III, BI, FIM, BBS, and SFR were included as independent variables, and 95% CI were reported along with regression parameters. The diagnostic utility of salivary biomarkers was evaluated using receiver operating curve (ROC) analysis. The area under the curve (AUC) and the cut-off point, characterized by the highest sensitivity and specificity, were calculated.

## Results

### Clinical Characteristics

Clinical characteristics of the groups are shown in **Table 2**. Thirty patients with stroke in the subacute phase were divided into two groups according to the stroke type: hemorrhagic and ischemic. There are no significant differences between both studies and control groups. Only functional and cognitive performance measured by the BI, FIM, BBS, and ACE III scores were significantly lower in stroke patients than controls. Oral examination and saliva collection were carried through between 30 and 35 days since the occurrence of stroke.

**Table 2:** Clinical characteristics of the studies and control groups.

		Group			p-Value			
		C	HS	IS	ANOVA	HS vs. C	IS vs. C	IS vs. HS
		*n* = 30	*n* = 15	*n* = 15				
Sex	Male *n* (%)	15 (50)	7 (46.66)	7 (46.66)	ND			
	Female *n* (%)	15 (50)	8 (53.33)	8 (53.33)				
Age *		63.07 ± 10.74 [59.06 - 67.08]	64.53 ± 8.123 [60.04 - 69.03]	61.6 ± 12.97 [54.42 - 68.78]	0.7586	0.9032	0.9032	0.7377
Education	Primary *n* (%)	2 (6.66)	1 (6.66)	1 (6.66)	> 0.9999			
	Vocational *n* (%)	15 (50)	7 (46.66)	6 (40)				
	Secondary *n* (%)	9 (30)	6 (40)	6 (40)				
	University *n* (%)	4 (13.33)	1 (6.66)	2 (13.33)				
Place of residence	Urban center *n* (%) *	10 (33.33)	6 (40)	7 (46.66)	> 0.9999			
	Small town *n* (%) *	8 (26.66)	4 (26.66)	3 (20)				
	Rural area or small village *n* (%) *	12 (40)	6 (40)	6 (40)				
*Cognitive and physical functional status*								
ACE III *		97.47 ± 1.48 [96.91 - 98.02]	69.47 ± 25.04 [55.6 - 83.33]	61.47 ± 22.33 [49.1 - 73.84]	< 0.0001	< 0.0001	< 0.0001	0.3929
BI*		20 ± 0 [20 - 20]	10.73 ± 4.166 [8.426 - 13.04]	10.47 ± 3.62 [8.46 - 12.47]	< 0.0001	< 0.0001	< 0.0001	0.9615
FIM*		125.2 ± 0.68 [125 - 125.5]	81.8 ± 34.16 [62.88 - 100.7]	83.47 ± 33.48 [64.93 - 102]	< 0.0001	< 0.0001	< 0.0001	0.9798
BBS*		55.53 ± 0.51 [55.34 - 55.72]	31.53 ± 18.91 [21.06 - 42.01]	28.47 ± 17.6 [18.72 - 38.21]	< 0.0001	< 0.0001	< 0.0001	0.7899
*Comorbidities*								
Diabetes *n* (%)		13 (43.33)	7 (46.66)	6 (40)	> 0.9999			
Hypertension *n* (%)		16 (53.33)	8 (53.33)	8 (53.33)	> 0.9999			
Arteriosclerosis *n* (%)		13 (43.33)	7 (46.66)	7 (46.66)	> 0.9999			
Limb thrombosis *n* (%)		4 (13.33)	2 (13.33)	2 (13.33)	> 0.9999			
Atrial fibrillation *n* (%)		5 (16.66)	2 (13.33)	3 (20)	> 0.9999			
*Drugs*								
< 5 drugs/day *n* (%)		16 (53.33)	7 (46.66)	6 (40)	> 0.9999			
≥ 5 drugs/day *n* (%)		14 (46.66)	8 (53.33)	9 (60)				

Results were analyzed using ANOVA analysis of variance followed by Tukey’s post hoc test; ACE III, Addenbrooke’s Cognitive Examination III; BBS, the Berg Balance Scale; BI, Barthel Index; C, Control group (n = 30); FIM, functional independence measure; HS, hemorrhagic stroke group (n = 15); IS, ischemic stroke group (n = 15); n, number of patients; ND, no data.

*Expressed as mean ± standard deviation (SD) [95% confidence interval (95% CI)].

### Salivary Gland Function

The hemorrhagic stroke group showed significantly lower SFR in SWS (-31%), and significantly lower TPC both in NWS (-19%) and SWS (-29%) regarding the control group. Patients after ischemic stroke incidents also presented significantly lower SFR in SWS (-24%), and TPC both in NWS (-27%) and SWS (-23%) relative to the control group (**Table 3**).

**Table 3:** Salivary gland function and stomatological characteristics of the studies and control groups.

		Group			p-Value			
		C	HS	IS	ANOVA	HS vs. C	IS vs. C	IS vs. HS
		*n* = 30	*n* = 15	*n* = 15				
SFR (mL/min)	NWS*	0.34 ± 0.09 [0.31 - 0.37]	0.39 ± 0.26 [0.24 - 0.52]	0.47 ± 0.25 [0.34 - 0.61]	0.1028	0.7393	0.084	0.4339
	SWS*	0.91 ± 0.26 [0.82 - 1.01]	0.63 ± 0.25 [0.5 - 0.77]	0.69 ± 0.29 [0.54 - 0.87]	0.0022	0.0039	0.0335	0.7732
TPC (μg/mL)	NWS*	1121 ± 188.4 [1050 - 1191]	904.5 ± 246.6 [768 - 1041]	817 ± 1 68.7 [723.5 - 910.4]	< 0.0001	0.0033	< 0.0001	0.4587
	SWS*	1240 ± 166.4 [1178 - 1303]	883.6 ± 250 [745.1 - 1022]	952.4 ± 225.4 [827.6 - 1077]	< 0.0001	< 0.0001	0.0001	0.6295
DMFT*		24.63 ± 7.25 [21.92 - 27.34]	22 ± 9.67 [16.64 - 27.36]	24.27 ± 3.84 [22.14 - 26.4]	0.5123	0.494	0.9862	0.674
GI*		0.74 ± 0.79 [0.44 - 1.04]	0.91 ± 0.77 [0.49 - 1.33]	0.6 ± 0.61 [0.26 - 0.94]	0.522	0.7598	0.8128	0.4906
PlI*		1.28 ± 1 [0.89 - 1.67]	1.45 ± 0.94 [0.93 - 1.96]	1.01 ± 0.9 [0.51 - 1.51]	0.464	0.8551	0.6607	0.4402

Results were analyzed using ANOVA analysis of variance followed by Tukey’s post hoc test; C, Control group (n = 30); DMFT, dental caries experience; GI, Gingival Index; HS, hemorrhagic stroke group (n = 15); IS, ischemic stroke group (n = 15); n, number of patients; NWS, non-stimulated whole saliva; PlI, Plaque Index; SFR, salivary flow rate; SWS, stimulated whole saliva; TPC, total protein content.

*Expressed as mean ± standard deviation (SD) [95% confidence interval (95% CI)].

### Stomatological Examination

The dental characteristics of the study group in comparison to the control group were shown in **Table 3**. Oral hygiene and periodontal status did not differ between groups.

### Salivary Ischemia Biomarkers

The standard colorimetric method failed to assess XO activity in saliva samples. Therefore, XO activity was measured fluorometrically using a commercial Amplex^®^ Red Xanthine/Xanthine Oxidase Assay Kit.

The group of patients suffering hemorrhagic stroke demonstrated significantly higher XO activity in NWS (+14%), XO specific activity in NWS (+45%) and SWS (+62%), H_2_O_2_ concentration in SWS (+20%), and UA concentration in NWS (+31%) concerning control group. The patients who suffered from hemorrhagic stroke shown significantly higher XO activity in NWS (+36%), XO specific activity in NWS (+89%) and SWS (+52%), XO output in NWS (+87%), H_2_O_2_ concentration in NWS (+109%) and SWS (+30%), and UA concentration in NWS (+73%) in comparison to the control group. Significantly differences between the ischemic and hemorrhagic stroke groups were observed in the case of the following parameters: XO activity (+20%), XO specific activity (+31%), XO output (+49%), H_2_O_2_ concentration (+65%), and UA concentration, all only in NWS (+32%) (**Table 4**).

**Table 4:** Salivary ischemia biomarkers of the studies and control groups.

		Group			p-Value			
		C	HS	IS	ANOVA	HS vs. C	IS vs. C	IS vs. HS
		*n* = 30	*n* = 15	*n* = 15				
XO activity (μU/mL)	NWS*	41.24 ± 6.61 [38.77 - 43.71]	46.99 ± 7.12 [43.05 - 50.93]	56.17 ± 7.75 [51.88 - 60.46]	< 0.0001	0.0325	< 0.0001	0.0021
	SWS*	38.37 ± 8.23 [35.29 - 41.44]	40.67 ± 8.74 [35.83 - 45.52]	42.99 ± 7.57 [38.79 - 47.18]	0.2039	0.6488	0.1851	0.7218
XO specific activity (nU/mg protein)	NWS*	37.99 ± 9.6 [34.4 - 41.57]	55.06 ± 14.56 [47 - 63.13]	71.94 ± 20.24 [60.73 - 83.15]	< 0.0001	0.0009	< 0.0001	0.0051
	SWS*	31.25 ± 6.8 [28.71 - 33.79]	50.59 ± 20.73 [39.11 - 62.07]	47.35 ± 12.89 [40.21 - 54.48]	< 0.0001	< 0.0001	0.0007	0.7751
XO output (μU/min)	NWS*	14 ± 3.96 [12.52 - 15.48]	17.62 ± 11.67 [11.15 - 24.08]	26.22 ± 14.65 [18.11 - 34.33]	0.0009	0.4701	0.0006	0.0474
	SWS*	34.93 ± 11.63 [30.59 - 39.28]	25.82 ± 12.12 [19.11 - 32.54]	29.75 ± 12.86 [22.63 - 36.88]	0.0562	0.0522	0.3697	0.6473
H_2_O_2_ concentration (nmol/L)	NWS*	239.7 ± 82.36 [208.9 - 270.5]	302.7 ± 72.37 [262.6 - 342.8]	500.9 ± 85.86 [453.3 - 548.4]	< 0.0001	0.7467	< 0.0001	< 0.0001
	SWS*	291.8 ± 77.43 [262.9 - 320.7]	351.1 ± 56.04 [320.1 - 382.1]	377.9 ± 89.92 [328.1 - 427.7]	0.0015	0.0438	0.0021	0.6024
UA concentration (μmol/L)	NWS*	58.83 ± 13.3 [53.86 - 63.8]	77.15 ± 23.25 [64.27 - 90.02]	102 ± 35.51 [82.37 - 121.7]	< 0.0001	0.0391	< 0.0001	0.0125
	SWS*	63.4 ± 18.34 [56.55 - 70.24]	57.87 ± 27.49 [42.65 - 73.1]	65.38 ± 31.49 [47.95 - 82.82]	0.6781	0.7571	0.9644	0.6804

ANOVA, analysis of variance; C, Control group (n = 30); H_2_O_2_, hydrogen peroxide; HS, hemorrhagic stroke group (n = 15); IS, ischemic stroke group (n = 15); n, number of patients; NWS, non-stimulated whole saliva; SWS, stimulated whole saliva; UA, uric acid; XO, xanthine oxidase.

*Expressed as mean ± standard deviation (SD) [95% confidence interval (95% CI)].

### Correlations

XO specific activity with UA concentration (*r* = -0.57, *p* = 0.001) and XO output with H_2_O_2_ level (*r* = -0.5, *p* = 0.005) were negatively correlated, and also H_2_O_2_ level was positively correlated with the BBS scale (*r* = 0.57, *p* = 0.001) in control group in NWS.

SWS in the control group showed a positive relationship only between H_2_O_2_ level and dynamic balance abilities in BBS (*r* = 0.57, *p* = 0.001).

Curiously enough, XO activity was positively correlated with UA (*r* = 0.49, *p* = 0.006) and H_2_O_2_ (*r* = 0.61, *p* < 0.001) concentrations in stroke group in NWS. XO specific activity presented positive interconnections with UA (*r* = 0.43, *p* = 0.017) and H_2_O_2_ (*r* = 0.49, *p* = 0.006) levels, and negative interconnections with ACE III (*r* = -0.71, *p* < 0.001), BI (*r* = -0.58, *p* = 0.001) and BBS (*r* = -0.61, *p* < 0.001) scores. Moreover, a positive relation between XO output and UA level (*r* = 0.46, *p* = 0.011) was shown. UA concentration corresponded positively with H_2_O_2_ concentration (*r* = 0.4, *p* = 0.027).

Interestingly, the study group showed negative correlations between UA concentration and dynamic balance abilities in BI (*r* = -0.42, *p* = 0.02), and BBS scales (*r* = -0.46, *p* = 0.011) in SWS.

**Figure 2:**
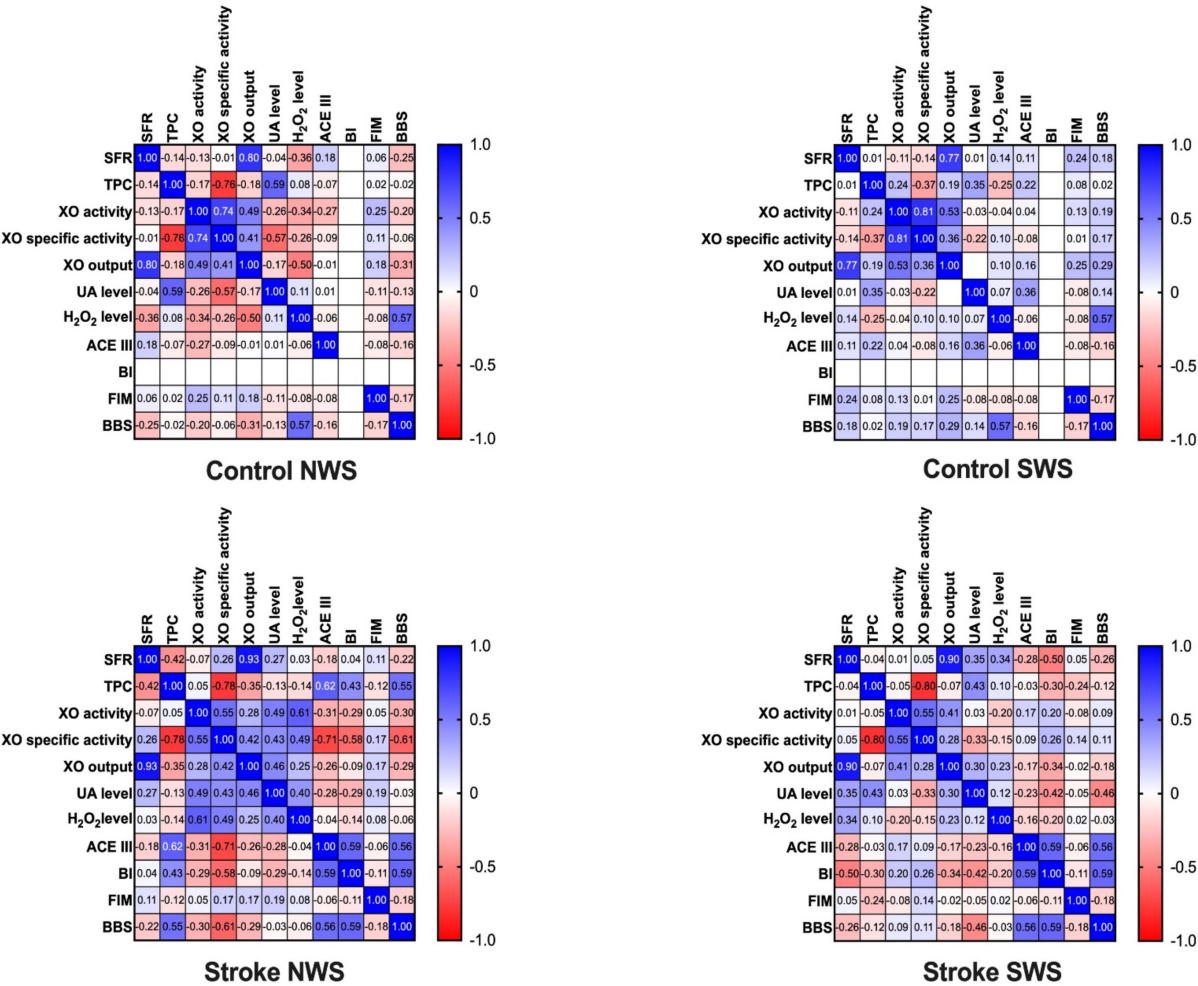
Correlations between salivary gland function, salivary ischemia biomarkers, and clinical parameters. ACE III, Addenbrooke’s Cognitive Examination III; BBS, the Berg Balance Scale; BI, Barthel Index; FIM, functional independence measure; H_2_O_2_, hydrogen peroxide; NWS, non-stimulated whole saliva; SFR, salivary flow rate; SWS, stimulated whole saliva; TPC, total protein content; UA, uric acid; XO, xanthine oxidase.

The above correlations were encapsulated in **Figure 2**.

### Regression Analysis

XO activity in NWS depends on stroke type, XO specific activity – stroke type and cognitive functions in ACE III, and XO output – stroke type and SFR. Furthermore, H_2_O_2_ and UA concentrations in NWS are affected by stroke type. In SWS, the variable influencing XO output is SFR, while UA level is affected by the FIM scale (**Table 5**).

**Table 5:** Multiple regression analysis of salivary ischemia biomarkers in all involved objects.

		NWS					SWS				
		XO activity	XO specific activity	XO output	H_2_O_2_ concentration	UA concentration	XO activity	XO specific activity	XO output	H_2_O_2_ concentration	UA concentration
β1: stroke type	Estimate	8.42	16.45	4.21	202.1	21.66	-0.008267	2.015	1.044	20.13	-13.56
	95% CI	2.49 - 14.35	8.67 - 24.23	0.47 - 7.95	137.2 - 267.1	0.09 - 43.23	-6.706 - 6.689	-11.4 - 15.43	-3.38 - 5.47	-55.59 - 95.85	-31.62 - 4.5
	p-Value	0.0074	0.0002	0.0291	< 0.0001	0.0491	0.998	0.7589	0.6302	0.5877	0.1341
β2: ACE III	Estimate	-0.02829	-0.2998	0.003936	1.435	-0.1792	-0.019	-0.03557	0.009605	-0.54	-0.4134
	95% CI	-0.19 - 0.14	-0.52 - -0.07	-0.1 - 0.11	-0.41 - 3.28	-0.79 - 0.43	-0.24 - 0.21	-0.48 - 0.41	-0.13 - 0.15	-3.07 - 1.99	-1.01 - 0.19
	p-Value	0.7317	0.0101	0.9397	0.122	0.5517	0.8623	0.8712	0.8944	0.6634	0.1706
β3: BI	Estimate	-0.3106	-1.223	-0.4194	-10.09	-3.855	-0.4503	0.4518	-0.2671	3.151	-2.186
	95% CI	-1.38 - 0.76	-2.63 - 0.18	-1.09 - 0.25	-21.83 - 1.64	-7.75 - 0.04	-1.67 - 0.76	-1.99 - 2.89	-1.07 - 0.54	-10.63 - 16.93	-5.48 - 1.1
	p-Value	0.5542	0.0848	0.212	0.0884	0.0522	0.4526	0.7055	0.4994	0.6407	0.1823
β4: FIM	Estimate	0.0008364	0.03768	0.01982	0.1577	0.1575	-0.009847	0.08176	-0.009425	-0.3358	-0.3762
	95% CI	-0.09 - 0.09	-0.08 - 0.16	-0.03- 0.07	-0.84 - 1.15	-0.18 - 0.49	-0.11 - 0.09	-0.12 - 0.29	-0.07 - 0.05	-1.5 - 0.83	-0.65 - -0.09
	p-Value	0.985	0.5199	0.4821	0.7463	0.3347	0.845	0.4209	0.777	0.5567	0.0101
β5: BBS	Estimate	-0.07378	-0.1856	-0.001744	0.4655	0.7937	0.1786	0.361	0.103	-0.7422	0.3472
	95% CI	-0.29 - 0.14	-0.48 - 0.1	-0.14 - 0.13	-1.97 - 2.9	-0.01 - 1.6	-0.06 - 0.42	-0.13 - 0.85	-0.05 - 0.27	-3.52 - 2.03	-0.31 - 1
	p-Value	0.5	0.2018	0.9797	0.6969	0.0545	0.146	0.1425	0.2017	0.5855	0.2893
β6: SFR	Estimate	-3.899	12.23	50.6	54.62	43.15	0.2419	5.205	41.9	119.2	17.25
	95% CI	-16.44 - 8.64	-4.22 - 28.69	42.69 - 58.52	-82.83 - 192.1	-2.47 - 88.77	-14.65 - 15.14	-24.63 - 35.04	32.06 - 51.74	-49.16 - 287.6	-22.92 - 57.43
	p-Value	0.5264	0.1377	< 0.0001	0.4195	0.0626	0.9735	0.7215	< 0.0001	0.1565	0.3836

ACE III, Addenbrooke’s Cognitive Examination III; BBS, the Berg Balance Scale; BI, Barthel Index; CI, confidence interval; FIM, functional independence measure; H_2_O_2_, hydrogen peroxide; NWS, non-stimulated whole saliva; SFR, salivary flow rate; SWS, stimulated whole saliva; UA, uric acid; XO, xanthine oxidase.

### ROC Analysis

Results of ROC analysis were shown in **Table 6**. Only XO specific activity was significantly different between hemorrhagic stroke and control groups, both in NWS and SWS. Statistically significant differences between patients with ischemic stroke and healthy controls were presented by XO activity in NWS, XO specific activity in NWS and SWS, H_2_O_2_ concentration in NWS and SWS, and UA level in NWS. Patients with ischemic stroke were significantly differentiated from those with hemorrhagic by XO activity, XO specific activity, and H_2_O_2_ level, all of them only in NWS.

**Table 6:** ROC analysis of salivary ischemia biomarkers.

		Cut off	AUC	95% CI	Sensitivity %	95% CI	Specificity %	95% CI
HS vs. C								
XO activity	NWS	> 44.78	0.747	0.59 - 0.91	80	54.81% - 92.95%	66.67	48.78% - 80.77%
	SWS	> 40.56	0.593	0.41 - 0.78	60	35.75% - 80.18%	66.67	48.78% - 80.77%
XO specific activity	NWS	> 45.94	0.858	0.74 - 0.97	73.33	48.05% - 89.1%	83.33	66.44% - 92.66%
	SWS	> 37.54	0.824	0.67 - 0.99	80	54.81% - 92.95%	86.67	70.32% - 94.69%
XO output	NWS	< 14.18	0.502	0.28 - 0.73	53.33	30.12% - 75.19%	50	33.15% - 66.85%
	SWS	< 30.95	0.693	0.53 - 0.86	66.67	41.71% - 84.82%	63.33	45.51% - 78.13%
H_2_O_2_ concentration	NWS	> 251.6	0.704	0.56 - 0.86	66.67	41.71% - 84.82%	56.67	39.2% - 72.62%
	SWS	> 325.4	0.733	0.59 - 0.88	73.33	48.05% - 89.1%	70	52.12% - 83.34%
UA concentration	NWS	> 65.44	0.735	0.59 - 0.89	66.67	41.71% - 84.82%	66.67	48.78% - 80.77%
	SWS	< 57.01	0.609	0.42 - 0.8	66.67	41.71% - 84.82%	73.33	55.55% - 85.82%
IS vs. C								
XO activity	NWS	> 49.61	0.92	0.82 - 1	86.67	62.12% - 97.63%	93.33	78.68% - 98.82%
	SWS	> 38.78	0.649	0.47 - 0.82	66.67	41.71% - 84.82%	56.67	39.2% - 72.62%
XO specific activity	NWS	> 50.3	0.973	0.94 - 1	93.33	70.18% - 99.66%	90	74.38% - 96.54%
	SWS	> 36.77	0.871	0.74 - 1	86.67	62.12% - 97.63%	83.33	66.44% - 92.66%
XO output	NWS	> 17.46	0.747	0.55 - 0.94	73.33	48.05% - 89.1%	83.33	66.44% - 92.66%
	SWS	< 32.65	0.607	0.43 - 0.79	53.33	30.12% - 75.19%	56.67	39.2% - 72.62%
H_2_O_2_ concentration	NWS	> 357.6	0.998	0.99 - 1	100	79.61% - 100%	96.67	83.33% - 99.83%
	SWS	> 328.5	0.762	0.61 - 0.92	66.67	41.71% - 84.82%	73.33	55.55% - 85.82%
UA concentration	NWS	> 68.82	0.873	0.74 - 1	86.67	62.12% - 97.63%	80	62.69% - 90.49%
	SWS	< 58.37	0.538	0.34 - 0.73	60	35.75% - 80.18%	66.67	48.78% - 80.77%
IS vs. HS								
XO activity	NWS	> 49.08	0.836	0.68 - 0.99	86.67	62.12% - 97.63%	73.33	48.05% - 89.1%
	SWS	> 40.87	0.551	0.34 - 0.77	53.33	30.12% - 75.19%	46.67	24.81% - 69.88%
XO specific activity	NWS	> 59.75	0.764	0.6 - 0.93	66.67	41.71% - 84.82%	66.67	41.71% - 84.82%
	SWS	< 46.92	0.516	0.3 - 0.73	53.33	30.12% - 75.19%	53.33	30.12% - 75.19%
XO output	NWS	> 20.09	0.684	0.5 - 0.9	66.67	41.71% - 84.82%	66.67	41.71% - 84.82%
	SWS	> 26.62	0.591	0.38 - 0.8	66.67	41.71% - 84.82%	53.33	30.12% - 75.19%
H_2_O_2_ concentration	NWS	> 392.6	0.96	0.9 - 1	93.33	70.18% - 99.66%	86.67	62.12% - 97.63%
	SWS	> 368.5	0.6	0.39 - 0.82	60	35.75% - 80.18%	66.67	41.71% - 84.82%
UA concentration	NWS	> 76.44	0.724	0.54 - 0.91	73.33	48.05% - 89.1%	66.67	41.71% - 84.82%
	SWS	> 53.19	0.587	0.38 - 0.8	66.67	41.71% - 84.82%	53.33	30.12% - 75.19%

AUC, the area under the curve; C, Control group (n = 30); CI, confidence interval; H_2_O_2_, hydrogen peroxide; HS, hemorrhagic stroke group (n = 15); IS, ischemic stroke group (n = 15); NWS, non-stimulated whole saliva; SWS: stimulated whole saliva; UA, uric acid; XO, xanthine oxidase.

**Figure 3:**
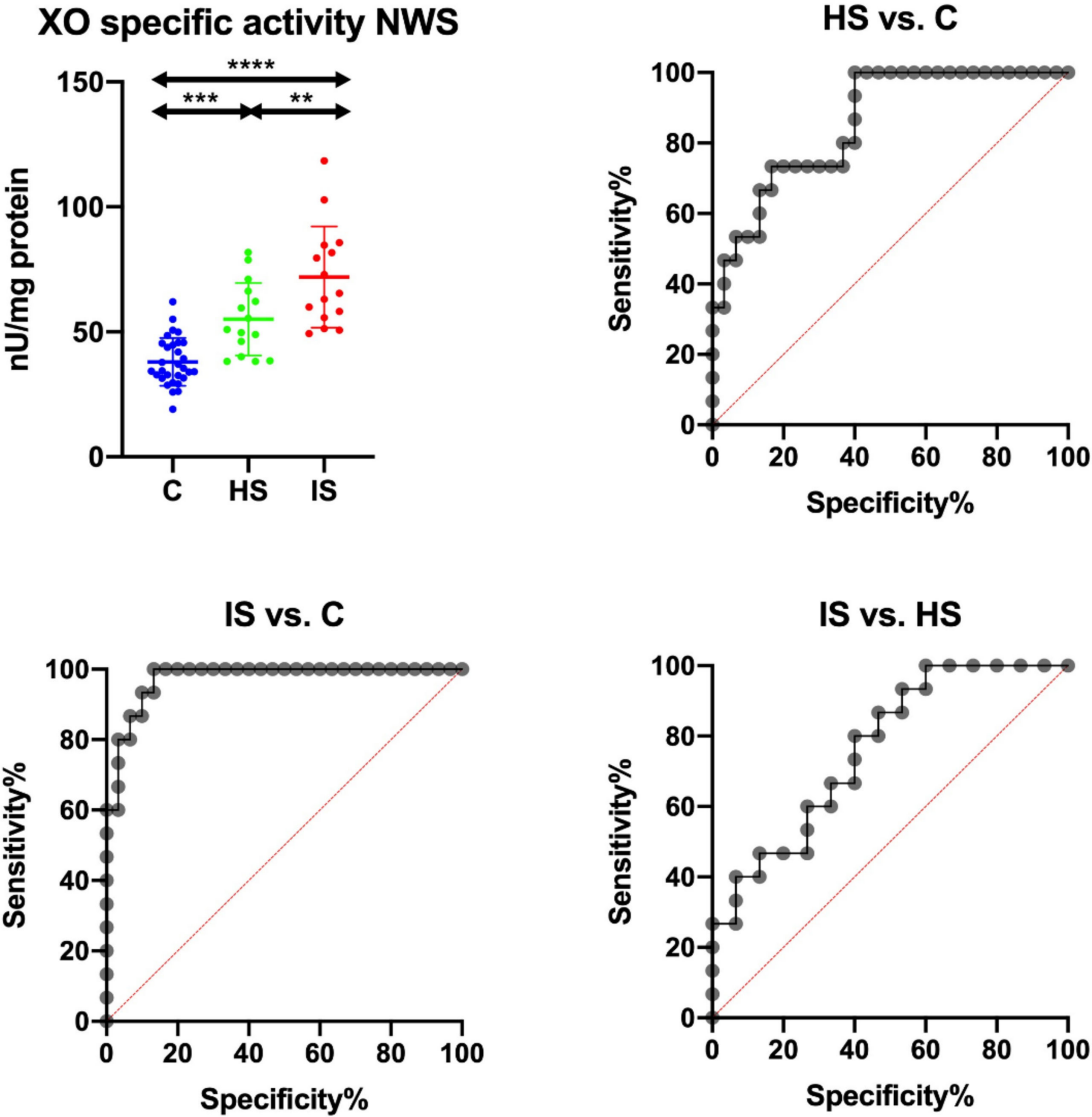
Results of ROC analysis for salivary XO specific activity in NWS. C, Control group (*n* = 30); HS, hemorrhagic stroke group (*n* = 15); IS, ischemic stroke group (*n* = 15); NWS, non-stimulated whole saliva; XO, xanthine oxidase. Differences statistically significant at: **p < 0.005; ***p < 0.0005; ****p < 0.0001.

Summarizing, the specific activity of XO in NWS is of particular diagnostic utility. This biomarker differentiated with high sensitivity and specificity hemorrhagic and ischemic strokes from control subjects. Moreover, it differentiates between both stroke types (**Table 6** and **Figure 3**).

### Clinical Utility of Salivary XO

**Figure 4:**
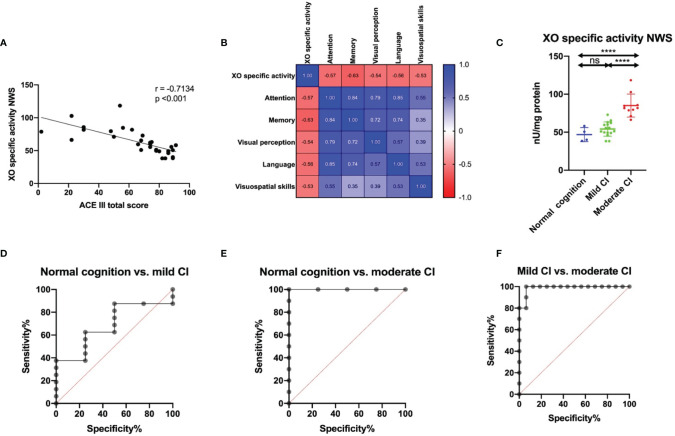
Correlation between salivary XO specific activity and cognitive function in ACE III scale **(A)**, correlations between XO specific activity and several parameters of ACE III score **(B)**; results of ROC analysis for salivary XO specific activity in relation to cognitive function status in ACE III scale in NWS **(C–F)**. ACE III, Addenbrooke’s Cognitive Examination III; mild CI, mild cognitive impairment; moderate CI, moderate cognitive impairment; NWS, non-stimulated whole saliva; XO, xanthine oxidase. Differences statistically significant at ****p < 0.0001. ns, non significance.

XO specific activity in NWS correlates negatively with ACE III total score (*r* = -0.71, *p* < 0.001) and its several cognitive abilities: attention and orientation (r = -0.57. p = 0.001), memory (r = -0.63, p < 0.001), visual perception (r = -0.54, p = 0.002), language (r = -0.56, p = 0.001) and visuospatial skills (r = -0.53, p = 0.003) (**Figures 4A, B**).

XO specific activity was significantly higher in stroke patients with moderate cognitive impairment compared with mild cognitive decline and subjects with normal cognitive function (**Figure 4C**). ROC analysis also confirms the clinical relevance of this biomarker. XO specific activity in NWS differentiates with high accuracy and specificity between moderate and mild cognitive impairment and healthy subjects (**Table 7** and **Figures 4C–F**).

**Table 7:** Results of ROC analysis for salivary XO specific activity in relation to cognitive function status in ACE III scale in NWS.

	Cut off	AUC	95% CI	Sensitivity %	95% CI	Specificity %	95% CI
Normal cognition vs. mild CI	> 50.79	0.688	0.42 - 0.95	62.5	38.64% - 81.52%	75	30.06% - 98.72%
Normal cognition vs. moderate CI	> 62.22	1	1 - 1	100	72.25% - 100%	100	51.01% - 100%
Mild CI vs. moderate CI	> 65.86	0.988	0.95 - 1	100	72.25% - 100%	93.75	71.67% - 99.68%

AUC, the area under the curve; CI, confidence interval; mild CI, mild cognitive impairment; moderate CI, moderate cognitive impairment.

## Discussion

Vascular brain diseases are one of the most common causes of death and disability worldwide. Despite significant progress in understanding stroke pathogenesis, the number of cases is still increasing (1, 66, 67). Therefore, laboratory biomarkers of stroke are sought to allow rapid and non-invasive diagnostics (15). Although diagnosis is based primarily on clinical examination and CT scans, it is not always possible to rule out conditions mimicking strokes, such as subdural or epidural hematoma, brain tumors, craniocerebral or cervical spine injury, infections (meningitis, encephalitis, brain abscess), seizures, migraine complications, and metabolic disturbances. Moreover, the sensitivity of CT in newly diagnosed ischemic stroke is less than 30-35%, which, in the absence of widespread availability of MRI and CT perfusion, indicates the need to search for new diagnostic strategies (12, 14). The ideal stroke biomarker should differentiate between ischemic and hemorrhagic stroke, have high sensitivity and specificity (at least 75-85%), capture disease dynamics/treatment effectiveness, and be easily quantifiable (68). In addition, the collection of biological material should be non-invasive, uncomplicated, and inexpensive. Therefore, saliva has become of increasing interest in the diagnosis of neurovascular diseases (69). Saliva collection is stress-free, requires no specialized medical personnel, and allows continuous monitoring of the patient’s condition through non-invasive sampling multiple times per day (18, 32).

The main factors leading to ischemic brain injury are inhibition of ATP production, excitotoxicity, inflammation, and cerebral oxidative/nitrosative stress. Indeed, lack of energy substrates disrupts depolarization of the neuronal membrane and increases intracellular levels of Na^+^, Cl^-^ and Ca^2+^ ions. This leads to activation of Ca^2+^-dependent enzymes such as protein kinase C (PKC), phospholipase A2, and other cellular proteases initiating neuronal apoptosis and necrosis. Simultaneously, there is a conversion of xanthine dehydrogenase to XO, which is crucial in post-stroke complications. The substrate for the enzyme is hypoxanthine (a breakdown product of ATP), which accumulates in the ischemic brain. When O_2_ is delivered under reperfusion, XO causes the conversion of hypoxanthine to xanthine accompanied by the release of superoxide radicals(O_2_-• ) *via* reduction of molecular oxygen. Subsequently O_2_-• induces the formation of more toxic ROS (e.g., H_2_O_2_) and stimulates the production of inflammatory mediators (24, 70, 71). However, XO also catalyzes the conversion of xanthine to UA (**Figure 1**). In ischemic stroke animal models, cerebral XO activity correlates with infarct volume and severity of neurological complications, thus postulating the use of XO in the identification/differentiation of the disease. Because assessment of XO activity in the blood is not diagnostically relevant (72), we were the first to investigate the usefulness of salivary XO in stroke patients.

We demonstrated that XO activity is significantly higher in NWS of ischemic stroke patients compared to hemorrhagic stroke and healthy subjects. However, statistically important differences were not observed in SWS. Indeed, the composition of stimulated saliva depends not only on the salivary gland but also on the environmental stimuli. Under resting conditions, 2/3 of the saliva is produced by the submandibular glands, whose filtrate generally reflects the composition of blood plasma. However, this ratio shifts during food/smell stimulation in favor of the parotid glands (52, 53, 73). These glands are also the primary source of antioxidants in the oral cavity, making SWS more protective against salivary oxidative stress (17, 74). The results of our previous studies support this. In SWS of stroke patients, we have shown higher activity/concentration of enzymatic and non-enzymatic antioxidants, which is an adaptive response to ROS overproduction in the parotid glands. These facts may explain the lack of differences in XO activity in SWS, especially if the concentration of enzyme substrates (xanthine and hypoxanthine) is very low in saliva samples. SWS is also much more dilute than NWS, making it less useful in laboratory medicine (33). Indeed, the main limitation of diagnostic applicability of saliva is the low biomarker concentration/activity compared to other bioliquids and tissues (75). Also in our study, salivary XO activity assessed by a standard enzyme assay was below the detection level in both NWS and SWS. However, using the Amplex Red xanthine/XO fluorometric method, XO can be detected at levels as low as 0.1 mU/mL. The use of a commercial kit has particular advantages in diagnostics, as it allows the comparison of results obtained in different laboratories.

The biomarkers found in saliva can be divided into compounds produced in the salivary glands and those outside of them (33, 74, 76). The ability to pass into saliva depends on the mechanism of transport and occurs *via* intracellular (diffusion, filtration, facilitated transport, active transport) or extracellular routes. Since most salivary proteins have a molecular weight between 20 and 60 kDa (low molecular weight proteins) (77), XO can migrate into saliva through ultrafiltration or damaged cellular membranes (78). XO is a homodimer composed of two subunits with an approximate molecular weight of 150-155 kDa (79). During BBB injury, XO can penetrate the brain into the circulation and then into the oral cavity. The salivary glands are very well vascularized resulted in a very efficient secretion of many substances into saliva (52). This may explain the higher correlation of many biomarkers between brain and NWS compared to blood (33). It may also result from a common developmental origin, or it may simply reflect the more significant variability of XO in the saliva (46). It should not be forgotten that XO can also be produced in the salivary glands (79). However, in some stroke patients, salivary gland dysfunction manifests as decreased salivary secretion (hyposalivation) or subjective dryness of the oral mucosa (xerostomia) (62, 80). Since the activity/concentration of salivary biomarkers is highly dependent on salivary gland function, we standardized XO measurement on total protein content and salivary flow rate. Our study indicates that XO activity should be standardized to total protein concentration, as with other salivary enzymes. Specific XO activity (nU/mg protein) better differentiates ischemic from hemorrhagic stroke and from healthy subjects.

The brain is particularly vulnerable to oxidation because it uses more than 20% of O_2_ supplied to the body (81). Neurons also show a very unfavorable surface-to-volume ratio, a high content of unsaturated fatty acids, and low efficiency of enzymatic antioxidant systems (82). Therefore, the main cause of brain damage during ischemia and reperfusion is the increased formation of ROS by XO (42). The superoxide radical generated by XO is enzymatically reduced to H_2_O_2_, with the simultaneous conversion of xanthine to uric acid. Although UA has strong antioxidant properties, in high concentrations, it also has a robust pro-oxidant effect. UA can generate free radicals by reaction with peroxynitrite or alkylate cellular biomolecules disrupting their structure and function (40). Thus, increased synthesis/release of proinflammatory cytokines, chemokines, and growth factors promotes neuronal apoptosis and necrosis under XO overexpression (83). In the acute phase of stroke, the BBB is disrupted, causing many biomarkers to infiltrate the blood and saliva (50). In our study, UA and H_2_O_2_ levels were significantly higher in stroke patients’ saliva than controls, whereas they did not differentiate between stroke types. Although XO activity correlated strongly with uric acid and hydrogen peroxide levels in stroke cases, their source in saliva may also be diffusion from blood plasma (UA) or exposure to environmental factors (84). Indeed, the oral cavity is the only place in the body exposed to many pro-oxidant agents such as diet, xenobiotics, oral microbiota, dental procedures, and materials (85–87).

An essential part of our study was also to evaluate the saliva’s usefulness for assessing XO activity in stroke patients. For this purpose, we used ROC curves, which are a graphical relationship between a test’s sensitivity and specificity. Of all the parameters evaluated, the specific activity of XO has the best clinical utility. We have shown that this biomarker differentiates ischemic stroke from hemorrhagic stroke (AUC: 0.764) and controls (AUC: 0.973) with very high sensitivity (IS vs. HS: 66.67%; IS vs. C: 93.33%) and specificity (IS vs. HS: 66.67%; IS vs. C: 90%). It is also noteworthy that XO specific activity correlates negatively with cognitive impairment according to ACE III scale, postural balance in BBS scale and performance in living activities using BI scale. Importantly, these relationships were observed only in the NWS of stroke patients. Unfortunately, we do not have clinical data on stroke location, brain infarct volume, and severity of neurological symptoms, making further clinical studies necessary. Although this undoubtedly represents limitations of the study, we are the first to demonstrate the utility of salivary XO in the differential diagnosis of stroke and for assessing patients’ functional status.

Stroke is the most common cause of cognitive impairment in people over 65 years old. Cognitive deficits include all areas of daily functioning affecting treatment and rehabilitation outcomes and quality of patient’s life (88). In our study, XO specific activity correlated highly negatively with cognitive abilities in the ACE III score. In detail, this biomarker was adversely associated with attention and orientation (r = -0.57, p = 0.001), memory (r = -0.63, p = 0.0002), visual perception (r = -0.54, p = 0.002), language (r = -0.56, p = 0.001) and visuospatial functions (r = -0.53, p = 0.003). ACE III is a comprehensive screening tool for cognitive function assessment, useful for early detection of cognitive impairment, differential diagnosis of dementia, and monitoring of the disease progression (54). Therefore, in the next step, we divided stroke patients into three subgroups based on the severity of cognitive decline: normal cognition (100-89 in the ACE III), mild cognitive impairment (88-61 in the ACE III), and moderate cognitive dysfunction (< 61 in the ACE III) (89, 90). We have demonstrated that XO specific activity differentiates with high accuracy (100%) and specificity (93.75%) between stroke patients with mild to moderate cognitive decline (AUC = 0.988). Thus, salivary XO assessment may be a potential screening tool for a comprehensive neuropsychological evaluation. Unfortunately, this parameter did not distinguish cognitively mild patients from those without cognitive impairment, which may be due to the small number of patients.

The ideal diagnostic biomarker should be detectable at an early stage of the disease. Our study involves patients in the acute phase of stroke (30-35 days after the incident), and therefore, it is essential to evaluate salivary XO activity in newly diagnosed cases. Since ischemia-reperfusion is most severe at this time, assessment of XO activity may have even greater diagnostic value (91).

In conclusion, our study demonstrates the utility of salivary xanthine oxidase in the differential diagnosis of stroke. The biological material for assessing XO activity should be non-stimulated saliva, and the results must be standardized to total protein content. We have shown that XO specific activity distinguishes, with very high sensitivity and specificity, between ischemic and hemorrhagic strokes and controls, as well as patients with mild and moderate cognitive impairment. Currently, there is no generally available and sensitive diagnostic test for stroke, which is a major limitation in its early diagnosis as well as its treatment. Salivary XO may be the first potential biomarker used in the differential diagnosis of stroke and to assess the functional status of patients.

Our study also has some limitations. Although appropriate statistical tests determined the sample size calculation, further studies on a larger patient population are needed. It is also necessary to evaluate XO activity in cases with periodontal and other oral and systemic diseases, which may affect the parameters assessed in saliva. It would also be interesting to perform a more extended follow-up of the patients, to investigate the possible correlation between evolution of XO salivary levels and the improvement of cognitive and physical functions, during the recovery after the acute phase of the stroke. To assess biomarker specificity, evaluation of salivary XO activity in patients with other neurological diseases [e.g., small vessel disease and transient ischemic attack (TIA)] is also essential.

## Data Availability Statement

The original contributions presented in the study are included in the article/supplementary material. Further inquiries can be directed to the corresponding author.

## Ethics Statement

The studies involving human participants were reviewed and approved by Local Ethics Committee at the Poznan University of Medical Sciences. The patients/participants provided their written informed consent to participate in this study.

## Author Contributions

MM: conceptualized, did laboratory determinations, performed statistical analysis, interpreted data, did performance of the graphic part of the manuscript, wrote the manuscript, reviewed the article, final approval of the version to be published, coordination of the project. MN: did laboratory determinations, interpreted data. AZ: conceptualized, did laboratory determinations, reviewed the article, final approval of the version to be published. GB: did laboratory determinations. PG: performed clinical examination, qualified patients to the study, collected material. KH: performed clinical examination, qualified patients to the study, collected material. KG: conceptualized, did laboratory determinations, reviewed the article, final approval of the version to be published. All authors contributed to the article and approved the submitted version.

## Funding

This work was supported by the Medical University of Bialystok, Poland (Grant No. SUB/1/DN/22/002/3330).

## Conflict of Interest

The authors declare that the research was conducted in the absence of any commercial or financial relationships that could be construed as a potential conflict of interest.

## Publisher’s Note

All claims expressed in this article are solely those of the authors and do not necessarily represent those of their affiliated organizations, or those of the publisher, the editors and the reviewers. Any product that may be evaluated in this article, or claim that may be made by its manufacturer, is not guaranteed or endorsed by the publisher.
